# Should We Report 15q11.2 BP1-BP2 Deletions and Duplications in the Prenatal Setting?

**DOI:** 10.3390/jcm9082602

**Published:** 2020-08-11

**Authors:** Idit Maya, Sharon Perlman, Mordechai Shohat, Sarit Kahana, Shiri Yacobson, Tamar Tenne, Ifaat Agmon-Fishman, Reut Tomashov Matar, Lina Basel-Salmon, Rivka Sukenik-Halevy

**Affiliations:** 1 Recanati Genetic Institute, Rabin Medical Center, Petah Tikva 49100, Israel; iditma@clalit.org.il (I.M.); kahanas@gmail.com (S.K.); shiricob@gmail.com (S.Y.); ifaat.agmon@gmail.com (I.A.-F.); rtomashov@gmail.com (R.T.M.); LinaB@clalit.org.il (L.B.-S.); 2 Sackler School of Medicine, Tel Aviv University, Tel Aviv 69978, Israel; drsharonperlman@gmail.com (S.P.); mshohat1@gmail.com (M.S.); 3 Ultrasound Unit, Helen Schneider Women’s Hospital, Rabin Medical Center, Petach Tikva 49100, Israel; 4 Genetic Institute, Maccabi Health medicinal organization, Rehovot, and Bioinformatics Unit, Cancer Research center, Chaim Sheba Medical Center, Tel-Hashome 52621, Israel; 5 Genetic Institute, Meir Medical Center, Kfar Saba 28164, Israel; tamar.tenne@gmail.com; 6 Felsenstein Medical Research Center, Rabin Medical Center, Petah Tikva 49100, Israel; 7 Pediatric Genetics Unit, Schneider Children’s Medical Center, Petah Tikva 49100, Israel

**Keywords:** chromosomal microarray, phenotype, BP1-BP2, 15q11.2, deletions, duplications, penetrance

## Abstract

Copy number variations of the 15q11.2 region at breakpoints 1-2 (BP1-BP2) have been associated with variable phenotypes and low penetrance. Detection of such variations in the prenatal setting can result in significant parental anxiety. The clinical significance of pre- and postnatally detected 15q11.2 BP1-BP2 deletions and duplications was assessed. Of 11,004 chromosomal microarray tests performed in a single referral lab (7596 prenatal, 3408 postnatal), deletions were detected in 66 cases: 39 in prenatal tests (0.51%) and 27 in postnatal tests (0.79%). Duplications were detected in 94 cases: 62 prenatal tests (0.82%) and 32 postnatal tests (0.94%). The prevalence of deletions and duplications among clinically indicated prenatal tests (0.57% and 0.9%, respectively) did not differ significantly in comparison to unindicated tests (0.49% and 0.78%, respectively). The prevalence of deletions and duplications among postnatal tests performed for clinical indications was similar to the prevalence in healthy individuals (0.73% and 1% vs. 0.98% and 0.74%, respectively). The calculated penetrance of deletions and duplications over the background risk was 2.18% and 1.16%, respectively. We conclude that the pathogenicity of 15q11.2 BP1-BP2 deletions and duplications is low. Opting out the report of these copy number variations to both clinicians and couples should be considered.

## 1. Introduction

Chromosomal microarray (CMA) testing is currently recommended as a first-tier test for individuals with unexplained developmental delay/intellectual disability, autism spectrum disorders, and multiple congenital anomalies [1]. In the prenatal setting, CMA analysis has replaced conventional karyotyping and became the primary test for prenatal diagnosis of congenital anomalies [2].

A major issue with CMA, especially in in the prenatal setting, is the challenges this test imposes in terms of determining the pathogenicity of certain findings and predicting the phenotype or the likelihood of medical and/or neurocognitive problems. It is clear that chromosomal syndromes differ not only in the type and severity of phenotypic characteristics but also in the variability of the phenotypic spectra and the likelihood of familial versus de novo findings [3]. Moreover, in some cases, the phenotype is affected by more than one copy number variation (CNV), as well as by the gender of the carrier and by other genetic variations and environmental factors [3]. The well-known chromosome region 15q11.2 is contiguous to the Prader–Willi/Angelman region. This region is prone to copy-number variations predisposed by several low copy repeats (LCRs), which can promote non-allelic, homologous recombination (NAHR) genomic rearrangements [4,5]. There are five known, recurring breakpoint regions (BP1-BP5) [4]. Prader–Willi and Angelman syndromes are related to the absence of gene expression from both parents in the 15q11.2-q13 region. Among the known mechanisms are deletions either on the paternal copy (causing Prader–Willi syndrome) or on the maternal copy (causing Angelman syndrome). The deletion class is typically subdivided into Type 1 and Type 2 based on their proximal breakpoints (BP1-BP3 and BP2-BP3, respectively). Several studies have shown that individuals with the larger deletion (BP1 to BP3) have more severe neurodevelopmental symptoms as compared to those with the typical, smaller deletions (BP2 to BP3) [6,7,8,9]. The region between BP1 and BP2 encompasses four highly conserved genes: *TUBGCP5, NIPA1, NIPA2* and *CYFIP1*. These genes are expressed in the central nervous system and are not subject to imprinting [10]. CNVs involving this region pose a significant challenge when detected in the prenatal setting as they have been reported in affected individuals in healthy family members of the affected proband [11] and in family members with phenotypes of varying severity [1,2]. Most of the CNVs in this region are inherited either maternally or paternally [3,11]. Deletions were reported in individuals with developmental delay, motor and speech impairments, schizophrenia, epilepsy, behavioral problems, attention deficit hyperactivity disorder (ADHD), obsessive-compulsive behavior, dysmorphism, malformations and an unusually happy expression [11,12,13,14,15,16,17]. Unaffected carriers were reported to have decreased cognitive abilities and general measures of functioning [18]. For these reasons, many refer to these CNVs as carrying an increased risk for a neurocognitive phenotype or as a region related to a variable phenotype with low penetrance.

Data regarding the clinical significance and related phenotypes of duplications of this region are less comprehensive. The reported phenotypes include dysmorphic features, developmental delay, autism, major behavior problems, attention deficit disorder/ attention deficit hyperactivity disorder, self-injury behaviors, neurological problems, and hypotonia [13,19].

This study assessed the prevalence and penetrance and clinical significance of BP1-BP2 deletions and duplications in the prenatal and postnatal settings and in indicated versus unindicated tests for the purpose of determining their significance and relevance in the prenatal setting. Our aim was to promote a discussion regarding the issue of reporting these CNVs.

## 2. Materials and Methods

### 2.1. Data Collection

The cohort was based on 11,004 CMA tests performed at a single clinical laboratory from 2013 through 2016. This laboratory serves several medical centers and private facilities. All 15q11.2 BP1-BP2 deletions and duplication cases were retrieved from the database. Complete data regarding patient characteristics, patient and family history, ethnic origin, the indication for testing, and test results were collected from a genetic counselling summary letter as well as a lab requisition form.

In the prenatal setting, indications for testing included cases in which a fetal malformation or abnormality was detected by ultrasound. The control group consisted of cases in which CMA was performed in low risk pregnancies with no indication for testing and a normal anatomical fetal scan: increased risk for Down syndrome (advanced maternal age and abnormal biochemical screening) and maternal request for invasive testing.

For the cohort of deletions, testing was performed on amniotic fluid in 36 cases (18 direct testing without culturing) and on chorionic villus samples in three cases.

For the cohort of duplications, testing was performed on amniotic fluid in 57 cases (31 cases of direct testing without culturing), on chorionic villus samples in two cases, on fetal blood in two cases and in one case on fetal tissue.

Postnatal cases included individuals with a variety of medical problems, including neurodevelopmental disorders, autism, congenital malformations, and combined medical problems. The control group consisted of healthy family members of individuals with a CNV other than BP1-BP2 deletion or duplication. Testing of family members in our lab was performed using CMA. Data regarding the clinical phenotype of these family members was obtained by the referring medical geneticist. Only family members with no known abnormal phenotype were used as controls. Cases in which testing was performed for BP1-BP2 deletion or duplication were excluded.

Some of the deletions in this cohort were reported and analyzed in a previous study [20] however, the group of postnatal tests performed in healthy family members of individuals with CNV other than 15q11.2 BP1-BP2 were not. The duplications in this cohort were not previously analyzed.

The study was approved by the RMC Institutional Ethics Review Board (0231-15-RMC).

### 2.2. Methods

#### 2.2.1. Chromosomal Microarray Analysis (CMA)

Chromosomal microarray analysis was performed using Human OmniExpress-24 v1.0 BeadChip (Illumina Inc., San Diego, CA, USA), which contains 716,503 genome-wide markers at an average spacing of 4 kb. It targets a minor allele frequency of 5%, as reported in the HapMap data. It includes SNPs within 10 kb of RefSeq genes, nonsynonymous SNPs (NCBI annotated), MHC/ADME SNPs, and sex chromosomes. DNA amplification, tagging, and hybridization were performed according to the manufacturer’s protocol, aided by the Tecan Freedom Evo (Tecan, Mannedorf, Switzerland). The array slides were scanned on an iScan Reader (Illumina, Inc., San Diego, CA, USA). All data collected were evaluated using Illumina Genome Studio v2011.1 software (Illumina, Inc., San Diego, CA, USA) and genome build GRCh37/hg19. Data were analyzed using Nexus Copy Number 7.5 (BioDiscovery, El Segundo, CA, USA). CNV classification was based on the American College of Medical Genetics and Genomics (ACMG) and the Clinical Genome Resource (ClinGen) 2020 guidelines [21].

#### 2.2.2. Analysis of Additional CNVs (Second Hits)

The presence of additional CNVs (second hits) was assessed in our cohort of cases with BP1-BP2 deletions and duplications. Each additional CNV detected was subjected to a recent revision by an expert in CMA analysis (I.M.), and its classification was determined based on current knowledge.

#### 2.2.3. Statistical Analysis

Four cohorts were analyzed separately: prenatal and postnatal 15q11.2 BP1-BP2 deletions and prenatal and postnatal 15q11.2 BP1-BP2 duplications. T-test and Fisher’s Exact Test were applied to test the between group differences. The indications for testing and specific characteristics were analyzed. A *p*-value of less than 0.05 was considered statistically significant. Python statistics library version 3.5.1 (scipy.stats) (Anaconda Inc, San Francisco, CA, USA) was used for statistical analysis.

#### 2.2.4. Calculation of Penetrance

Penetrance was calculated for deletions and duplications as follows: The prevalence of abnormal phenotypes in the general population included in the calculations was 5.12%, based on previously reported disease frequency used for penetrance calculations [22,23]. The frequency of 15q11.2 BP1-BP2 deletions and duplications in the normal population was calculated according to the control groups of healthy individuals and those who had unindicated prenatal testing. The penetrance was calculated as the ratio between affected individuals with 15q11.2 BP1-BP2 deletion/duplication (number of affected individuals in the population times the frequency of the CNV found in our cohort) and the prevalence of these deletions and duplications in the general population.

#### 2.2.5. Power Analysis

The number of cases needed to detect a threefold increase in the incidence of prenatally detected deletions and duplications (power of 0.8) was 3170 and 1978, respectively (for a two-fold increase; 9538 and 5964, respectively).

The number of cases needed to detect a threefold increase in the incidence of postnatally detected deletions and duplications (power of 0.8) was 1568 and 2088, respectively (for a two-fold increase, 4732 and 6270, respectively).

Data regarding the study cohort of deletions and duplications is presented in Table S1.

## 3. Results

### 3.1. 15q11.2 BP1-BP2 Deletions

#### 3.1.1. In Prenatal Diagnosis

Among 39 cases of 15q11.2 BP1-BP2 deletions, 14 (prevalence 0.57%) were detected in tests performed for a clinical indication, and 25 (prevalence 0.49%) were detected in tests performed without a clinical indication. (Table 1).

The indications for testing among the prenatally detected deletions are listed in Table 2.

The prevalence of 15q11.2 BP1-BP2 deletions was similar for prenatal testing with or without an indication (0.57% vs. 0.49%, respectively; *p* = 0.6).

Data regarding the cohort of pregnancies in which 15q11.2 BP1-BP2 deletions were detected are presented in Table 3. Average parental age, distribution of fetal sex, average length of the deletion, and the prevalence of additional copy number variations did not differ significantly between indicated and unindicated cases. (*p* = 0.75).

#### 3.1.2. In Postnatal Diagnosis

Among 27 cases of 15q11.2 BP1-BP2 deletions, 19 were detected in individuals with an abnormal phenotype, and 8 were detected in individuals tested for an abnormal CMA result in a family member (prevalence 0.73% and 0.98%, respectively, (*p* = 0.497), Table 1).

The indications for postnatal testing are listed in Table 4. There was no difference in the male to female ratio between deletions detected in individuals with an abnormal phenotype and healthy family members of individuals with abnormal CMA results (percentage of males: 55% vs. 45%, *p* = 0.77). The length of the deletion in the two groups was similar (754,606 ± 168,754 vs. 788,586 ± 182,970, *p* = 0.6).

Overall, no statistically significant difference was found in the rate of 15q11.2 BP1-BP2 deletions in unindicated prenatal tests and postnatal 15q11.2 BP1-BP2 deletions found in affected individuals (*p* = 0.2).

### 3.2. 15.q11.2 BP1-Bp2 Duplications

#### 3.2.1. In Prenatal Diagnosis

Among 62 cases of 15q11.2 BP1-BP2 duplications, 22 (prevalence 0.9%) were detected in tests performed for an indication and 40 (prevalence 0.78%) were detected in tests performed without a specific indication (Table 1). The indications for testing in the cases of prenatally detected duplications are listed in Table 2. Three cases with duplications detected prenatally underwent termination of pregnancy due to one or more major malformation. The prevalence of 15q11.2 BP1-BP2 duplications did not differ significantly between indicated prenatal tests and unindicated tests (0.9% vs. 0.78%, *p* = 0.59).

The distribution of fetal sex was similar between indicated and unindicated cases (*p* = 0.8). The average length of the deletion was similar for the 2 groups, as was the prevalence of additional copy number variations. Average paternal age was similar between cases in which CMA testing was performed with or without an indication. However, average maternal age was significantly higher in the group tested without an indication.

#### 3.2.2. In Postnatal Diagnosis

Among 32 cases of 15q11.2 BP1-BP2 duplications, 26 (prevalence 1%) were detected in individuals with an abnormal phenotype and 6 (prevalence 0.74%) in individuals tested for of an abnormal CMA result in a family member (*p* = 0.67) (Table 1).

The indications for postnatal testing are listed in Table 4. The male to female ratio between duplications detected in individuals with an abnormal phenotype and healthy family members of individuals with abnormal CMA results was similar (percentage of males: 59% vs. 67%; *p* = 1). The length of the duplications was similar in both groups (767,197 ± 221,388 vs. 773,197 ± 228,165; *p* = 0.95).

Overall, there was no statistically significant difference in the rate of 15q11.2 BP1-BP2 duplications in prenatal unindicated tests and postnatal duplications found in affected individuals (*p* = 0.36).

### 3.3. Additional CNVs (Second Hits)

Within the cohort of patients with deletions, 8 cases had additional CNVs (20.5% of cases with deletions) (Table S2), and in one case, two CNVs were detected. Altogether, there were 5 deletions and 4 duplications: Three were classified as pathogenic and 5 classified as VUS (an additional CNV classified as VUS was present along with a CNV classified as pathogenic). The rate of pathogenic second hits between indicated and unindicated tests (both prenatal and postnatal) was close to reach statistical significance (*p* = 0.059). The rate of all second hits (both pathogenic and VUS) was not statistically different (*p* = 0.64) between indicated and unindicated cases (both prenatal and postnatal) with deletions.

Within the cohort of patients with duplications, 10 cases had additional CNVs (16.1% of cases with duplications) (Table S3); in one case, both trisomy 18 and an additional CNV were detected. Altogether, there were 6 deletions and 4 duplications: five were classified as pathogenic or likely pathogenic and 5 classified as VUS (an additional CNV classified as VUS was present along with trisomy 18). The rate of pathogenic second hits and of all second hits (both pathogenic and VUS) between indicated and unindicated tests (both prenatal and postnatal) was not statistically different (*p* = 0.12, *p* = 0.36, respectively).

### 3.4. Penetrance

Based on our data, the calculated penetrance of 15q11.2 BP1-BP2 deletions and duplications was 7.3% and 6.28%, respectively.

## 4. Discussion

The data presented here indicates that 15q11.2 BP1-BP2 deletions and duplications are common among affected and unaffected populations, with a prevalence of 0.6% for deletions and 0.85% for duplications. The prevalence of deletions and duplications was not statistically different between cases tested for an abnormal phenotype compared to testing in low risk pregnancies.

The phenotypes related to CNVs are known for their variability, incomplete penetrance and wide phenotypical spectra, even among members of the same family. Factors suggested as modifiers or contributors to the clinical presentation of CNV carriers include the presence of other CNVs, single-nucleotide variants, the sex of the carrier, and environmental or epigenetic factors [3,24,25,26]. According to our data, the calculated penetrance of deletions and duplications was 7.3% and 6.28%, respectively. This calculation was based on a 5.12% frequency of abnormal phenotypes in the general population. This indicates a minimal addition of 2.18% and 1.16% over the background risk. If we would have used a lower figure for the frequency of affected individuals in the population, the calculated penetrance would be accordingly lower. The penetrance of 15q11.2 BP1-BP2 deletions was previously estimated at 5% to10% [20]. Girirajan et al. [3] showed that 15q11.2 BP1-BP2 deletions are inherited in more than 90% of cases and that in approximately 15% of cases, additional CNVs are detected in affected individuals. Butler et al. reviewed 200 cases reported in the literature and concluded that 15q11.2 BP1-BP2 deletions are de novo in only 5–22% [27]. In our cohort, the rate of additional CNVs (both pathogenic and VUS) was 20.5% for cases with deletions and 16.1% for cases with duplications. Interestingly, the sex of the carrier and the rate of second hits between indicated and unindicated tests (both prenatal and postnatal) was not statistically different.

The prevalence of CNVs involving 15q11.2 BP1-BP2 in our study was not significantly different from that reported by Burnside et al. [19] who analyzed a large cohort of subjects with 15q11.2 BP1-BP2 CNVs, with similar phenotypic features. The prevalence of CNVs involving 15q11.2 BP1-BP2 was 0.86% of all individuals referred for microarray analysis, including 69 subjects with deletions (0.41% of total) and 77 with duplications (0.45% of total). Common phenotypes noted in these cases included autism, developmental, motor and language delays, and behavioral problems. Parental studies demonstrated phenotypically normal carriers in several instances and mildly affected carriers in others.

Previous studies have shown that 15q11.2 BP1-BP2 deletions are linked to a modest risk for schizophrenia [17,28] behavioral disturbances [11] developmental and language delays [11,19], and epilepsy [29]. A recent meta-analysis performed on previously published data reported 15q11.2 deletions to be associated with a 4.3 point decrease in IQ scores and an ORs and respective frequencies for intellectual disabilities, schizophrenia and epilepsy of 1.7 (3.4%), 1.5 (2%), and 3.1 (2.1%), respectively. The authors used 15q11.2 duplications as a neutral control variant [17]. Stefansson et al. [30] reported that 15q11.2 BP1-BP2 deletions have only a small impact on results of neuropsychological tests but are strongly associated with a history of difficulties in learning mathematics and reading, even after adjusting for IQ. Interestingly, MR imaging performed in deletion carriers showed abnormalities in brain structure in a pattern consistent with that observed during first-episode psychosis in schizophrenia (a reduced volume of grey matter in the perigenual anterior cingulate cortex and the left insula, reduced white matter in the temporal lobes bilaterally, and increased volume of the corpus callosum). In addition, they reported a nominally significant reduction in fecundity in individuals with deletions of 15q11.2 BP1-BP2. Van der Meer et al. [31] assessed cortical and subcortical morphology and cognition in carriers of 15q11.2 BP1-BP2 CNVs and reported a lower surface area, thicker cortex, and a smaller nucleus accumbens in deletion carriers. Deletion carriers performed significantly lower than controls in on all cognition tasks [31].

In a previous study, our lab analyzed the clinical significance of 20 known CNVs in prenatal and postnatal samples, including the 15q11.2 BP1-BP2 deletion, which was categorized as a low penetrance (less than 10%) CNV [20]. The frequency of low-penetrance CNVs in this study was not significantly different among prenatal unindicated tests, cases of prenatal high-risk tests (prenatally detected congenital malformations), and postnatal cases with unexplained developmental delay, ID, autism spectrum disorders, or multiple congenital anomalies. In comparison, high-penetrance CNVs were significantly more frequent in postnatal testing and in high-risk pregnancies. We concluded that a low-penetrance CNV alone should not be considered pathogenic, should not be used to predict phenotype, and should not be reported to the parents in the prenatal setting. In our previous study, no calculation of the penetrance was done, and data regarding the prevalence of duplications was not included.

Mohan et al. [32] recently published a study that assessed the association between four phenotypes (developmental delay, dysmorphic features, autism group of disorders, and epilepsy/seizures) and BP1-BP2 deletions and duplications. Their cohort included 262 patients with deletions and 215 with duplications that were detected among 51,462 patients referred for genetic testing at two centers. The only association found in this cohort was between deletions and dysmorphism. The calculated penetrance for this phenotype based on the frequency of the deletions among controls reported as by Rosenfeld et al. [22] was low (3.8%). Kendall at al. [18] studied unaffected carriers of 33 recurrent CNVs including deletions and duplications of 15q11.2 BP1-BP2, using tests for cognitive abilities and for general measures of functioning. They CNVs implicated in neurodevelopmental disorders, including schizophrenia, associated with cognitive deficits, even among unaffected individuals. The reduction in test performance, however, was modest for most CMVs. Interestingly, while the range of impairments in carriers of 15q11.2 the deletion was mild, it was larger than anticipated. The duplication was associated with a minimal decline in test performance. The penetrance indicating the cumulative risk of CNV carriers to develop schizophrenia, autism spectrum disorder, or intellectual disability was 8% for deletion carriers and 5% for carriers of duplications. Our cohort consisted of both prenatal and postnatal tests that were both indicated and unindicated. The penetrance calculation was done for any abnormal phenotype for both deletions and duplications.

Specific data regarding 15q11.2 BP1-BP2 duplications are scarce. Results of neurocognitive testing performed on 136 controls carrying the 15q11.2 BP1-BP2 duplication were comparable to those of population controls [30]. MRI imaging in duplication carriers showed reciprocal changes in exactly the same regions altered in deletion carriers, providing the first demonstration of allele-dose-dependent effects of CNVs on the structure of the human brain [30]. Van der Meer et al. [31] reported a lower cortical thickness in duplication carriers. These individuals however, performed similarly to noncarriers on cognitive tasks.

The issue of reporting deletions and duplications of the 15q11.2 BP1-BP2 region in the prenatal setting. This information can be extremely stressful for the future parents, and the phycological consequences during pregnancy and afterwards should not be underestimated. Our data suggest that the susceptibility for abnormal phenotypes related to these CNVs is not significant and that the risk related to these findings is minimal. The manner of classification of low-penetrant variants has raised considerable controversy among laboratories, and an option to define a category of “High-frequency low-penetrance” has been discussed [33,34]. In our lab, we classify the deletion as likely benign and the duplication as benign. It has been previously suggested that a low-penetrance CNV alone should not be reported to the parents in the prenatal setting [20]. Mohan et al. [32] suggested to provide an option for prenatal consent not to be informed about 15q11.2 BP1-BP2 deletions and duplications. However, the authors state that “unless such a consent is in place, it seems preferable to report the finding with a statement that these CNVs rarely cause a defect that requires a clinical action or reproductive decision”. Marshall et al. [17] recommend classifying the deletion as “pathogenic of mild effect size” and suggested that it is not worth discussing it in the developmental clinic or in a prenatal setting since it explains only a small proportion of the phenotypic variance in carriers. We suggest offering couples the option to opt out of the report regarding these CNVs as well as a list of other CNVs with similar clinical significance. In the case of 15q11.2 BP1-BP2 duplications, our data support the insignificance of these duplications as risk factors for neurocognitive phenotypes. Hence, we believe an even more liberal option where labs may decide not to report this finding at all in all cases. It is important that if couples are being reported regarding 15q11.2 BP1-BP2 deletions and duplications, they should receive comprehensive clinical information and counselling in order to give the correct perspective regarding such findings. We believe that generally, classification should not differ between the pre- and postnatal setting, as postnatal counselling frequently raises a discussion regarding pregestational and preimplantation diagnosis. Importantly, parental testing is not performed for these cases. We currently do not offer to opt out on reporting deletions in the postnatal setting. However, since the 15q11.2 BP1-BP2 deletions are considered as modifiers or second hits, their detection in an affected individual does not usually explain the phenotype and another genetic cause should be sought. The study of such additional hits is an extensive task, far beyond the scope of this study. In our cohort, the rate of pathogenic second hits was not different between indicated and unindicated tests (for both deletions and duplications); however, it was close to reaching statistical significance for deletions. Veltman et al. [35] hypothesized that the majority of second hits are probably not detectable even by very high-resolution arrays and that Exome and eventually whole-genome re-sequencing may well reveal a surprising number of additional contributing loci. Hashemi et al. [36] concluded that the term 15q11.2 (BP1-BP2) deletion “syndrome” be avoided.

Brain imaging in carriers of 15q11.2 BP1-BP2 deletions and duplications is significant for the assessment of the pathogenicity of such CNVs. At this point, prenatal data regarding fetal MR imaging in these cases are lacking. Future imaging studies may shed light on this issue.

This study assessed the prevalence of 15q11.2 BP1-BP2 CNVs in affected and unaffected populations in order to determine if the prevalence is significantly higher in unaffected populations. For prenatal cases, the unaffected group consisted of cases with no indication for testing while in postnatal cases the unaffected cohort included individuals reported as healthy. We did not perform any clinical assessment in postnatal cases, and we have no information regarding the cognition, behavior, mental health, and other phenotypes that are undetectable in the prenatal setting. Hence, the prenatal control group in not optimal and could be regarded as a cohort that can provide an assessment of the prevalence of these CNVs in the general population. While data regarding the prevalence of deletions in healthy and affected populations are extensive, data regarding the prevalence in prenatal tests are limited, and specifically, data regarding the duplication are scarce. The study was powered to show a threefold change in prevalence for deletions and duplications in both prenatal and postnatal tests but was underpowered for a two-fold change in prevalence. Based on the Odds ratios reported in the literature, it is not surprising that there was no statistical difference in the prevalence of the CNVs in the different groups. The study was limited by the lack of clinical assessment of each healthy and affected carrier of these CNVs. In addition, our information was based on the report submitted to the lab by the geneticist that referred the patient and not on our own assessments. Information regarding the postnatal phenotype among the cases detected prenatally is lacking. A future study that will formally assess prenatally detected cases may have significant clinical value and may provide useful information when interpreting and consulting cases with 15q11.2 BP1-BP2 deletions and duplications.

## 5. Conclusions

Our data show that 15q11.2 BP1-BP2 deletions and duplications are common findings among affected and unaffected populations, indicating their low pathogenicity and minimally increased risk for abnormal phenotypes. Hence, reporting these CNVs in the prenatal setting should be discussed with couples before testing. We suggest that opting out of reporting these CNVs both to clinicians as well as to couples should be considered.

## Figures and Tables

**Table 1:** The prevalence of deletions and duplications involving the 15q11.2 BP1-BP2 region.

Reason for Test	Number of Tests	Deletion N (%)	Duplication N (%)
Prenatal			
With indication ^†^	2447	14 (0.57)	22 (0.90)
Without indication ^‡^	5149	25 (0.49)	40 (0.78)
Total prenatal	7596	39 (0.51)	62 (0.82)
Postnatal			
Pathologic phenotype ^§^	2595	19 (0.73)	26 (1.00)
Family member with abnormal CMA ^§§^	813	8 (0.98)	6 (0.74)
Total postnatal	3408	27 (0.79)	32 (0.94)
Total tests performed	11004	66	94

^†^ Prenatal cases with indication included cases in which a fetal malformation or abnormality was detected by ultrasound. ^‡^ Prenatal cases without indication included cases in which Chromosomal microarray (CMA) was performed with no indication. These included cases in which invasive testing was done due to increased risk for Down syndrome (advanced maternal age, combination of soft signs, abnormal biochemical screen), as well as maternal request for invasive testing with no specific indication. ^§^ Postnatal cases with indication included individuals with a variety of medical problems, including neurodevelopmental disorders, autism, congenital malformations, and combined medical problems. ^§§^ Postnatal cases without indication included of healthy family members of individuals with a copy number variation (CNV) other than BP1-BP2 deletion or duplication.

**Table 2:** Indications for CMA testing in prenatally detected 15q11.2 BP1-BP2 copy number variations.

Indication	Cases with Deletion N (% ^†^)	Cases with Duplication N (% ^†^)
Isolated malformation	3 (21.4)	9 (40.1)
Combined malformations	1 (7.1)	1(4.5)
Soft sign	0	1 (4.5)
Malformation and a soft sign	0	0
Increased nuchal translucency ^‡^	8 (57.1)	2 (9.1)
Intrauterine growth restriction	0	5 (22.7)
Polyhydramnios	0	3 (13.6)
Macrosomia	0	1 (4.5)
Abnormal sonographic findings (not including soft signs)	2 (14.3) 0	0
Total	14	22

^†^ Percent out of indicated prenatal tests. ^‡^ Nuchal translucency >3 mm.

**Table 3:** 15q11.2 BP1-BP2 copy number variations detected prenatally.

Prenatal Cases	15q11.2 Deletion			15q11.2 Duplication		
	With Indication	Without Indication	*p*-Value	With Indication	Without Indication	*p*-Value
Number of cases (incidence)	14 (0.57%)	25 (0.49%)	0.60	22 (0.9)	40 (0.78)	0.59
Male fetus (%)	9 (60)	14 (53.8%)	0.75	13 (54.2)	23 (57.5)	0.8
Average maternal age (years ± SD)	32.2 ± 4.2	34.8 ± 4.5	0.18	33.5 ± 2.9	36.1 ± 4.3	0.05
Average paternal age (years ± SD)	35 ± 3.2	35.7 ± 3.9	0.69	35.9 ± 4.3	37.9 ± 6.4	0.38
Average length of CNV (bases ± SD)	743,998 ± 175,980	781,279 ± 172,492	0.51	803,456 ± 212,408	791,410 ± 219,739	0.83

**Table 4:** Indications for CMA testing in postnatally detected 15q11.2 BP1-BP2 copy number variations.

Indication	Cases with 15q11.2 Deletion (% ^†^)	Cases with 15q11.2 Duplication (% ^†^)
Intellectual disability (ID)	3 (15.7)	4 (15.4)
ID and additional medical problems	11 (57.9)	11 (42.3)
Autistic spectrum disorder	0	3 (11.5)
Medical problems not involving ID	3 (15.8)	0
Malformation	1 (5.2)	5 (19.2)
Other	1 (5.2)	3 (11.5)
Total	19	26

^†^ Percent of indicated postnatal tests.

## Supplementary Materials

The following are available online at https://www.mdpi.com/2077-0383/9/8/2602/s1, Table S1: Cases with 15q11.2 BP1-BP2 deletions and duplications, Table S2: Additional CNVs detected in the cohort of cases with 15q11.2 BP1-BP2 deletions, Table S3: Additional CNVs detected in the cohort of cases with 15q11.2 BP1-BP2 duplications.
