# Supplementary material for: Should We Report 15q11.2 BP1-BP2 Deletions and Duplications in the Prenatal Setting?
Source: J Clin Med. 2020 Aug 11;9(8):2602. doi: 10.3390/jcm9082602 (PMC7463673; doi:10.3390/jcm9082602)
Supplement: Supplementary file 1 [file jcm-09-02602-s001.pdf]

Table S1. Cases with 15q11.2 BP1-BP2 deletions and duplications

15q11 BP1-BP2 deletions		Gender	Sample Source	Indication for Chromosomal microarray (CMA)	CMA result	Type of copy number variation (CNV)	genomic region GRCH37/HG19	CNV length (base pairs)
**PRENATAL**								
**Prenatal with indication**	1	M	AF	NT=3.1mm, cervical cyst	Normal CMA	loss	chr15:22,701,317-23,272,733	571416
	2	F	AF	NT=3.1mm	pathogenic: loss 16p11.2 (0.6MB)	loss	chr15:22,701,317-23,272,733	571416
	3	M	CVS	Moderate TR, mild MR, cystic hygroma	Normal CMA	loss	chr15:22,752,019-23,272,733	520714
	4	M	AF	Elevated nuchal fold	Normal CMA	loss	chr15:22,299,434-23,272,733	973299
	5	M	AF	Left clubfoot	Normal CMA	loss	chr15:22,299,434-23,272,733	973299
	6	M	AF	Cleft lip	Normal CMA	loss	chr15:22,614,224-23,272,733	658509
	7	F	AF	NT=3.2mm	Normal CMA	loss	chr15:22,614,224-23,272,733	658509
	8	F	AF	NT=3mm	Normal CMA	loss	chr15:22,299,434-23,272,733	973299
	9	M	CVS	NT=3.1mm	Normal CMA	loss	chr15:22,614,224-23,272,733	658509
	10	M	AF	NT=3.4mm	NOS: del 18p11.23 (0.4 Mb)	loss	chr15:22,299,434-23,272,733	973299

	11	F	AF	IUGR severe, echogenic bowel, complicated heart malformation (TOF), SUA	Normal CMA	loss	chr15:22,522,310-23,272,733	750423
	12	F	AF	Ascites moderate, LLQ abdominal cysts, polyhydramnios AFI=255 mm	Normal CMA	loss	chr15:22,701,317-23,288,275	586958
	13	F	CVS	NT=3.5mm	Normal CMA	loss	chr15:22,614,224-23,272,733	658509
	14	M	AF	VSD	Normal CMA	loss	chr15:22,299,434-23,272,733	973299

**Prenatal without indication**	1	F	AF	Parental request	Normal CMA	loss	chr15:22,299,434-23,272,733	973299
	2	F	AF	Parental request	Normal CMA	loss	chr15:22,299,434-23,272,733	973299
	3	F	AF	Advanced maternal age	Normal CMA	loss	chr15:22,614,224-23,272,733	658509
	4	F	AF	Parental request	Normal CMA	loss	chr15:22,614,224-23,272,733	658509
	5	F	AF	Biochemical screen for Down syndrome: 1:35	NOS: gain 15q25.2 (0.89 MB)	loss	chr15:22,614,224-23,272,733	658509
	6	M	AF	Parental request	Normal CMA	loss	chr15:22,299,434-23,272,733	973299
	7	F	AF	Parental request	Normal CMA	loss	chr15:22,614,224-23,272,733	658509
	8	M	AF	Advanced maternal age	Normal CMA	loss	chr15:22,614,224-23,272,733	658509
	9	M	AF	Advanced maternal age	Normal CMA	loss	chr15:22,299,434-23,272,733	973299
	10	F	AF	Parental request	Normal CMA	loss	chr15:22,299,434-23,272,733	973299

	11	M	AF	Parental request	Normal CMA	loss	chr15:22,299,434-23,272,733	973299
	12	M	AF	Advanced maternal age	Normal CMA	loss	chr15:22,614,224-23,272,733	658509
	13	F	AF	Advanced maternal age	Normal CMA	loss	chr15:22,614,224-23,272,733	658509
	14	M	AF	Parental request	Normal CMA	loss	chr15:22,701,317-23,272,733	571416
	15	M	AF	Parental request	Normal CMA	loss	chr15:22,614,224-23,272,733	658509
	16	M	AF	Parental request	Normal CMA	loss	chr15:22,614,224-23,272,733	658509
	17	M	AF	Advanced maternal age	Normal CMA	loss	chr15:22,299,434-23,272,733	973299
	18	M	AF	Parental request	Normal CMA	loss	chr15:22,299,434-23,272,733	973299
	19	M	AF	Advanced maternal age	Normal CMA	loss	chr15:22,614,224-23,272,733	658509
	20	M	AF	Advanced maternal age	Normal CMA	loss	chr15:22,701,317-23,272,733	571416
	21	M	AF	Parental request	Normal CMA	loss	chr15:22,299,434-23,288,275	988841
	22	F	AF	Advanced maternal age	Normal CMA	loss	chr15:22,299,434-23,288,275	988841
	23	M	AF	Advanced maternal age	Normal CMA	loss	chr15:22,701,317-23,288,275	586958
	24	F	AF	Biochemical screen for Down syndrome: 1:141	Normal CMA	loss	chr15:22,701,317-23,288,275	586958
**POSTNATAL**								
	1	F	Blood	ID, ADHD	Normal CMA	loss	chr15:22,614,224-23,272,733	658509

**POSTNATAL with indication**	2	F	Blood	ID, dysmorphism, IUGR, abnormal brain MRI, bilateral hydronephrosis	Normal CMA	loss	chr15:22,614,224-23,272,733	658509
	3	F	Blood	Cleft and lip palate	Normal CMA	loss	chr15:22,299,434-23,272,733	973299
	4	M	Blood	ID, seizures	Normal CMA	loss	chr15:22,614,224-23,272,733	658509
	5	F	Blood	Hypotonia, VSD, macrocephaly, abnormal brain MRI	Normal CMA	loss	chr15:22,614,224-23,272,733	658509
	6	F	Blood	ID, dysmorphism, short stature	Normal CMA	loss	chr15:22,614,224-23,272,733	658509
	7	M	Blood	Abnormal brain MRI, microcephaly	Normal CMA	loss	chr15:22,614,224-23,272,733	658509
	8	M	Blood	ID, dysmorphism	Normal CMA	loss	chr15:22,299,434-23,272,733	973299
	9	M	Blood	ID	Normal CMA	loss	chr15:22,299,434-23,272,733	973299
	10	F	Blood	ID, hydrocephalus, Autism, right aortic arch, ASD	Normal CMA	loss	chr15:22,701,317-23,272,733	571416
	11	M	Blood	hypotonia, FTT, cryptorchidism	Normal CMA	loss	chr15:22,299,434-23,272,733	973299
	12	M	Blood	ID, seizures, microcephaly, cerebellar atrophy	Normal CMA	loss	chr15:22,299,434-23,272,733	973299
	13	M	Blood	ID, dysmorphism, pectus excavatum, frontal bossing	Normal CMA	loss	chr15:22,614,224-23,272,733	658509
	14	M	Blood	Mild ID, ADHD	Normal CMA	loss	chr15:22,299,434-23,272,733	973299
	15	F	Blood	ID, congenital heart malformation: Bicuspid aortic valve, coarctation of the aorta	Normal CMA	loss	chr15:22,701,317-23,288,275	586958

	16	F	Blood	ID, seizures, dysmorphism, macrocephaly	Normal CMA	loss	chr15:22,768,255-23,288,275	520020
	17	M	Blood	ID	pathogenic: loss 15q11.2 (6.5Mb)-AS/PWS recurrent loss	loss	chr15:22,614,224-23,272,733	658509
	18	F	Blood	Seizures, FTT	pathogenic: loss proximal 16p11.2 deletion syndrome (0.54Mb)(TBX6)	loss	chr15:22,299,434-23,272,733	973299
	19	M	Blood	ID	pathogenic: loss 16p11.2 (0.67 Mb)(TBX6), NOS: gain 7p21.3 (0.8Mb)	loss	chr15:22,614,224-23,288,275	674051
**POSTNATAL without indication**	1	M	Blood	daughter with seizures, FTT and pathogenic: loss 16p11.2 (0.54Mb)(TBX6)	Normal CMA	loss	chr15:22,299,434-23,272,733	973299
	2	F	Blood	Mother of fetus with clef lip palate and NOS: gain 18q12.3 (2.9Mb)	NOS: gain 18q12.3 (2.9Mb)	loss	chr15:22,299,434-23,272,733	973299
	3	F	Blood	Mother of fetus with NT=3.1mm and pathogenic: del 16p11.2 (0.6MB)	Normal CMA	loss	chr15:22,299,434-23,272,733	973299
	4	M	Blood	father of fetus with absent kidney and NOS: gain 2q11.2 (1.4 Mb)	Normal CMA	loss	chr15:22,701,317-23,272,733	571416
	5	F	Blood	Mother of fetus with NOS gain 16q24.3 (455 kb)	NOS: gain 16q24.3 (0.4Mb)	loss	chr15:22,701,317-23,272,733	571416
	6	F	Blood	Son with MR and NOS deletion 7q11.23(1.5Mb)	NOS: loss 7q11.23(1.5Mb)	loss	chr15:22,614,224-23,272,733	658509

	7	M	Blood	Mother of fetus with NOS: del 18p11.23 (0.4 Mb)	Normal CMA	loss	chr15:22,299,434-23,272,733	973299
	8	M	Blood	Father of fetus with pathogenic: del 1q21 (2.45MB)	Normal CMA	loss	chr15:22,614,224-23,272,733	658509
**15q11 BP1-BP2 duplications**		Gender	Sample Source	Indication for CMA	Results	TYPE OF 15q11 CNV	genomic region	CNV length (BP)
**PRENATAL**								
**Prenatal with indication**	1	F	AF	IUGR, short long bones	Normal CMA	gain	chr15:22,701,317-23,272,733	571416
	2	M	AF	Right clubfoot	Normal CMA	gain	chr15:22,448,234-23,272,733	824499
	3	M	AF	IUGR	Normal CMA	gain	chr15:22,299,434-23,272,733	973299
	4	M	AF	Polyhydramnios AFI=250 mm	Normal CMA	gain	chr15:22,701,317-23,272,733	571416
	5	F	AF	Polyhydramnios AFI=265 mm, decreased fetal movements	Normal CMA	gain	chr15:22,768,255-23,272,733	504478
	6	F	AF	Polyhydramnios	Normal CMA	gain	chr15:22,299,434-23,272,733	973299
	7	M	AF	VSD, asymmetry of heart ventricles	Normal CMA	gain	chr15:22,299,434-23,272,733	973299
	8	F	AF	IUGR, golf ball	Normal CMA	gain	chr15:22,299,434-23,272,733	973299
	9	M	AF	TOP due to multiple congenital anomalies	Normal CMA	gain	chr15:22,701,317-23,272,733	571416
	10	F	AF	NT=3.6mm	Normal CMA	gain	chr15:22,701,317-23,272,733	571416

	11	F	AF	Hypoplasia of mid phalanges, TR, CPC	Normal CMA	gain	chr15:22,299,434-23,272,733	973299
	12	F	AF	IUGR, SUA, golf ball	Normal CMA	gain	chr15:22,701,317-23,272,733	571416
	13	F	AF	Ventriculomegaly bilateral 10.4mm	Normal CMA	gain	chr15:22,299,434-23,272,733	973299
	14	M	AF	Bicuspid aortic valve, VSD	Normal CMA	gain	chr15:22,785,793-23,272,733	486940
	15	M	CVS	NT=4.1mm septeted cystic hygroma	pathogenic: trisomy 18 ; NOS: gain 19q13.31 (1Mb)	gain	chr15:22,299,434-23,272,733	973299
	16	F	AF	VSD muscular posterior	Normal CMA	gain	chr15:22,299,434-23,272,733	973299
	17	M	Fetal Blood	TOP due to MRI brain abnormal, septo-optic dysplasia, posterior fossa dilated 11mm, hypoplastic vermis	Normal CMA	gain	chr15:22,299,434-23,272,733	973299
	18	M	AF	Macrosomia (+3SD)	Normal CMA	gain	chr15:22,299,434-23,288,275	988841
	19	M	AF	Congenital heart malformation	Normal CMA	gain	chr15:22,299,434-23,288,275	988841
	20	M	Fetal tissue	Oligohydramnios, lower urinary tract obstruction	Normal CMA	gain	chr15:22,701,317-23,288,275	586958
	21	M	AF	Kidney agenesis (left)	Normal CMA	gain	chr15:22,299,434-23,288,275	988841
	22	F	AF	IUGR (-2SD)	Normal CMA	gain	chr15:22,299,434-23,288,275	988841
	1	M	AF	soft sign- cervical cyst	Normal CMA	gain	chr15:22,299,434-23,272,733	973299

	2	F	AF	AMA	Normal CMA	gain	chr15:22,299,434-23,272,733	973299
	3	F	AF	AMA	Normal CMA	gain	chr15:22,299,434-23,272,733	973299
	4	M	AF	AMA	Normal CMA	gain	chr15:22,836,555-23,272,733	436178
	5	M	AF	AMA	Normal CMA	gain	chr15:22,299,434-23,272,733	973299
	6	M	AF	AMA	Normal CMA	gain	chr15:22,299,434-23,272,733	973299
	7	F	AF	AMA	Normal CMA	gain	chr15:22,299,434-23,272,733	973299
	8	M	AF	AMA	Normal CMA	gain	chr15:22,614,224-23,272,733	658509
Prenatal without indication	9	M	AF	AMA	Normal CMA	gain	chr15:22,299,434-23,272,733	973299
	10	F	AF	AMA	Normal CMA	gain	chr15:22,841,135-23,272,733	431598
	11	M	AF	AMA	Normal CMA	gain	chr15:22,701,317-23,272,733	571416
	12	F	AF	AMA	Normal CMA	gain	chr15:22,299,434-23,272,733	973299
	13	M	AF	AMA	Normal CMA	gain	chr15:22,299,434-23,272,733	973299
	14	F	AF	AMA	Normal CMA	gain	chr15:22,788,062-23,272,733	484671
	15	M	AF	AMA	Normal CMA	gain	chr15:22,614,224-23,288,275	674051
	16	F	AF	AMA	Normal CMA	gain	chr15:22,299,434-23,288,275	988841
	17	M	AF	AMA	Normal CMA	gain	chr15:22,299,434-23,288,275	988841

18	M	AF	AMA	Normal CMA	gain	chr15:22,299,434-23,288,275	988841
19	M	AF	AMA	Normal CMA	gain	chr15:22,701,317-23,288,275	586958
20	F	AF	AMA	Normal CMA	gain	chr15:22,614,224-23,288,275	674051
21	M	AF	AMA, golf ball	Normal CMA	gain	chr15:22,299,434-23,288,275	988841
22	F	AF	Abnormal biochemical screen for Down syndrome	Normal CMA	gain	chr15:22,768,255-23,272,733	504478
23	M	AF	Parental request	Normal CMA	gain	chr15:22,701,317-23,272,733	571416
24	F	AF	Parental request	Normal CMA	gain	chr15:22,835,806-23,272,733	436927
25	M	AF	Parental request	Normal CMA	gain	chr15:22,299,434-23,272,733	973299
26	F	AF	Parental request	Normal CMA	gain	chr15:22,701,317-23,272,733	571416
27	M	AF	Parental request	Normal CMA	gain	chr15:22,299,434-23,272,733	973299
28	M	AF	Parental request	Normal CMA	gain	chr15:22,383,292-23,272,733	889441
29	M	AF	Parental request	Normal CMA	gain	chr15:22,701,317-23,272,733	571416
30	M	AF	Parental request	Normal CMA	gain	chr15:22,299,434-23,272,733	973299
31	F	AF	Parental request	Normal CMA	gain	chr15:22,299,434-23,272,733	973299
32	F	AF	Parental request	Normal CMA	gain	chr15:22,299,434-23,272,733	973299
33	M	AF	Parental request	Normal CMA	gain	chr15:22,299,434-23,272,733	973299

	34	F	AF	Parental request	Normal CMA	gain	chr15:22,299,434-23,272,733	973299
	35	F	AF	Parental request	Normal CMA	gain	chr15:22,299,434-23,272,733	973299
	36	M	AF	Parental request	Normal CMA	gain	chr15:22,785,793-23,272,733	486940
	37	F	AF	Parental request	NOS: gain Xq26.3 (0.5 Mb)	gain	chr15:22,701,317-23,272,733	571416
	38	M	AF	Parental request	Normal CMA	gain	chr15:22,798,916-23,272,733	473817
	39	M	AF	Parental request	Normal CMA	gain	chr15:22,299,434-23,272,733	973299
	40	F	CVS	Parental request	Normal CMA	gain	chr15:22,701,317-23,288,275	586958
POSTNATAL								
**POSTNATAL with indication**	1	F	Blood	Omphalocele, left hydronephrosis, PDA	normal	gain	chr15:22,299,434-23,272,733	973300
	2	M	Blood	Hypotonia, craniosynostosis	normal	gain	chr15:22,768,255-23,272,733	504478
	3	M	Blood	Congenital microcephaly, ID, agenesis of corpuscallosum, dysmorphism, FTT	normal	gain	chr15:22,299,434-23,272,733	973299
	4	M	Blood	Developmental delay (motor)	normal	gain	chr15:22,299,434-23,272,733	973299
	5	M	Blood	Azoospermia, high anal atresia, vesiculo urethral fistula	normal	gain	chr15:22,701,317-23,272,733	571416
	6	M	Blood	CDH, bilateral cryptorchidism	normal	gain	chr15:22,768,255-23,272,733	504478
	7	M	Blood	Aortic coarctation, hypopigmentary spots	normal	gain	chr15:22,299,434-23,272,733	973299

	8	F	Blood	Severe ID, hypotonia, abnormal brain MRI	normal	gain	chr15:22,299,434-23,272,733	973299
	9	F	Blood	Autistic spectrum disorder	normal	gain	chr15:22,701,317-23,272,733	571416
	10	F	Blood	ID	normal	gain	chr15:22,299,434-23,272,733	973299
	11	M	Blood	Abnormal ears and cleft lip and palate	normal	gain	chr15:22,841,135-23,272,733	431598
	12	M	Blood	Autistic spectrum disorder, dysmorphism, ID	normal	gain	chr15:22,299,434-23,272,733	973299
	13	M	Blood	ID, epilepsy	normal	gain	chr15:22,701,317-23,272,733	571416
	14	M	Blood	ID, short stature, hypothyroidism, Growth hormone abnormality	normal	gain	chr15:22,299,434-23,272,733	973299
	15	F	Blood	TGA	normal	gain	chr15:22,299,434-23,272,733	973299
	16	M	Blood	Autistic spectrum disorder	normal	gain	chr15:22,701,317-23,272,733	571416
	17	M	Blood	ID	normal	gain	chr15:22,299,434-23,272,733	973299
	18	F	Blood	ID, hypotonia, deafness	normal	gain	chr15:22,788,062-23,272,733	484671
	19	F	Blood	ID, dysmorphism	normal	gain	chr15:22,701,317-23,272,733	571416
	20	M	Blood	ID, dysmorphism	normal	gain	chr15:22,299,434-23,272,733	973299
	21	F	Blood	Mild ID	NOS: loss 19q13.2 (0.3 Mb)	gain	chr15:22,701,317-23,272,733	571416
	22	F	Blood	ID, dysmorphism	NOS: loss 15q13.3 (0.6 MB)	gain	chr15:22,299,434-23,272,733	973299

	23	M	Blood	Autistic spectrum disorder, Microcephaly, ADHD	pathogenic: gain 7q11.23 (1.1 Mb)	gain	chr15:22,299,434-23,272,733	973299
	24	F	Blood	Hypotonia, sister with NOS: loss 19q13.2 (0.3Mb)	NOS: loss 19q13.2 (0.3Mb)	gain	chr15:22,614,224-23,272,733	658509
	25	F	Blood	ID	NOS: gain 19q13.33 (0.6Mb)	gain	chr15:22,701,317-23,272,733	571416
	26	M	Blood	ID, congenital heart malformation TOF, abnormal brain MRI agenesis of corpus calloum, brother with similar phenotypes and pathogenic CMA del 15q13.3	pathogenic: loss 15q13.2-q13.3 (1.7Mb)	gain	chr15:22,299,434-23,272,733	973299
**POSTNATAL without indication**	1	M	Blood	Father of fetus with NOS: loss 7p12.1 (1.5Mb)	NOS: loss 7p12.1 (1.5Mb)	gain	chr15:22,701,317-23,272,733	571416
	2	M	Blood	Father of fetus with NOS: loss 10p13 (1.2MB)	normal parent	gain	chr15:22,299,434-23,272,733	973299
	3	M	Blood	Father of fetus with LP: gain 16p11.2 (0.6Mb) (TBX6)	normal parent	gain	chr15:22,299,434-23,272,733	973299
	4	M	Blood	Mother of child with autism and LP: loss 16p12.2 (0.6 Mb)	LP: del 16p12.2 (0.5 Mb)	gain	chr15:22,798,916-23,272,733	473817
	5	F	Blood	Mother of fetus with NT=4.1mm septeted cystic hygroma and trisomy 18 and NOS gain 19q13.31 (1 Mb)	Normal CMA	gain	chr15:22,299,434-23,272,733	973299
	6	F	Blood	Mother of fetus with NT= 3.2mm and Pathogenic: gain16p11.2 (0.7Mb) TBX6	Normal CMA	gain	chr15:22,614,224-23,288,275	674051

NT= Nuchal translucency, AF= Amniotic fluid, CVS= Chorionic villus sampling, TR-tricuspid regurgitation, MR- mitral regurgitation, IUGR=intrauterine growth restriction, TOF=tetralogy of fallot, SUA=single umbilical artery, LLQ- left lower quadrant, VSD=ventricular septal defect, DD= developmental delay, ADHD= attention

deficit hyperactivity disorder, ID= intellectual disability, ASD=atrial septal defect, FTT- failure to thrive, AFI=amniotic fluid index, CPC= choroid plexus cyst, SD=standard deviation, PDA=patent ductus arteriosus, CDH=congenital dislocation of hip.

Table S2. Additional CNVs detected in the cohort of cases with 15q11.2 BP1-BP2 deletions.

case	Additional CNV	Location	Classification	Indication for testing
**1**	gain 18q12.3 (2.9Mb)	chr18:37,528,230-40,438,159	VUS	Postnatal: healthy<br>Mother of a fetus with clef lip palate and a 18q12.3 gain (2.9Mb)
**2**	loss 18p11.23 (0.4 Mb)	chr18:7,753,507-8,218,209	VUS	Prenatal: elevated nuchal translucency (3.4 mm)
**3**	1. loss 16p11.2 (0.67 Mb)(TBX6)<br>2. gain 7p21.3 (0.8Mb)	1. chr16:29,631,159-30,289,846<br>2. chr7:13,173,144-13,967,577	1.Pathogenic- known recurrent microdeletion syndrome<br>2.VUS	Postnatal : Intellectual disability (ID)
**4**	gain 15q25.2 (0.89 MB),	chr15:84,881,977-85,772,391	VUS	Prenatal: no indication
**5**	loss 7q11.23(1.5Mb)	chr7:74,938,902-76,431,306	VUS	Postnatal healthy (Son with ID and a 7q11.23 loss (1.5Mb)
**6**	loss 15q11.2 (6.5Mb)	chr15:23,656,946-28,535,266	Pathogenic- prader willi/Angelman syndorome	Postnatal : ID
**7**	loss 16p11.2 (0.6MB)(TBX6)	chr16:29,649,915-30,281,111	Pathogenic- known recurrent microdeletion syndrome	Prenatal: increased nuchal translucency (3.1 mm)
**8**	gain 16q24.3 (0.4Mb)	chr16:89,654,641-90,109,773	VUS	Postnatal healthy:<br>Mother of fetus with a 16q24.3 gain (0.4Mb)

VUS- variants of unknown clinical significance.

Table S3. Additional CNVs detected in the cohort of cases with 15q11.2 BP1-BP2 duplications.

case	Additional CNV	location	Classification	Indication for testing
**1**	loss 15q13.3 (0.6 MB)	chr15:32,019,325-32,620,127	Likely pathogenic	Postnatal: Intellectual disability (ID) , dysmorphism
**2**	gain 7q11.23 (1.1 Mb)	chr7:72,722,981-74,138,603	Pathogenic	Postnatal:<br>Autism, Microcephaly , ADHD
**3**	1. trisomy 18 ;<br>2. gain 19q13.31 (1Mb)	1.chr18:1-78,077,248<br>2. chr19:44,670,104-45,764,916	1. Pathogenic:<br>2. VUS	Prenatal: nuchal translucency =4.1mm, cystic hygroma
**4**	loss 15q13.2-q13.3 (1.7Mb)	chr15:30,936,285-32,620,127	Pathogenic	Postnatal: ID, Tetralogy of Fallot, Agenesis of the corpus callous, brother with similar phenotype and similar 15q13.3 deletion
**5**	loss 19q13.2 (0.3Mb)	chr19:40,927,463-41,303,187	VUS	Postnatal: healthy
**6**	loss 19q13.2 (0.3 Mb)	chr19:40,927,463-41,303,187	VUS	Postnatal, mild ID, hypotonia, two sisters with ID and hypotonia, two parents with mild ID
**7**	gain 19q13.33 (0.6Mb)	chr19:48,026,884-48,597,532	VUS	Postnatal: ID
**8**	gain Xq26.3 (0.5 Mb)	chrX:134,266,696-134,779,230	VUS	Prenatal: no indication
**9**	loss 7p12.1 (1.5Mb)	chr7:51,848,824-53,363,895	VUS	Postnatal: healthy: father of fetus with 7p12.1 loss (1.5Mb)
**10**	loss 16p12.2 (0.5 Mb)	chr16:21,894,231-22,422,637	Likely pathogenic	Postnatal: healthy: daughter with autism and 16p12.2 deletion (0.5 Mb)

VUS- variants of unknown clinical significance.
